# Impaired Whole-Blood Fibrinolysis is a Predictor of Mortality in Intensive Care Patients

**DOI:** 10.1055/a-2270-7673

**Published:** 2024-03-28

**Authors:** Julie S. Brewer, Christine L. Hvas, Anne-Mette Hvas, Julie B. Larsen

**Affiliations:** 1 Department of Clinical Biochemistry, Thrombosis and Hemostasis Research Unit, Aarhus University Hospital, Aarhus, Denmark; 2 Department of Anaesthesiology and Intensive Care, Aarhus University Hospital, Aarhus, Denmark; 3 Department of Clinical Medicine, Aarhus University, Aarhus, Denmark; 4 Dean of the Faculty of Health, Aarhus University, Aarhus, Denmark

**Keywords:** blood coagulation tests, critical care, fibrinolysis, sepsis, thromboelastography

## Abstract

**Background** Altered fibrinolysis is considered to play a crucial role in the development of coagulopathy in sepsis. However, routine laboratory tests for fibrinolysis are currently very limited, and the impact of fibrinolytic capacity on clinical outcome is poorly investigated.

**Objectives** To assess whole-blood fibrinolysis in patients admitted to the intensive care unit (ICU) and compare fibrinolysis in sepsis patients with nonsepsis patients. Further, to investigate associations between fibrinolytic capacity and 30-day mortality and venous thromboembolism (VTE).

**Methods** This study was designed as a prospective cohort study. Adult ICU patients were included at the Aarhus University Hospital, Denmark. All patients had a blood sample obtained the morning after admission. A modified thromboelastometry (ROTEM®) analysis with tissue plasminogen activator (ROTEM®-tPA) was used to assess fibrinolysis. The primary endpoint was difference in ROTEM®-tPA lysis time between sepsis patients and nonsepsis patients.

**Results** ROTEM®-tPA revealed fibrinolytic impairment in sepsis patients ( *n* = 30) compared with nonsepsis ICU controls ( *n* = 129), with longer lysis time (median [interquartile range] 3,600 [3,352–3,600] vs. 3,374 seconds [2,175–3,600], *p* < 0.01), lower maximum lysis (23 [8–90] vs. 94% [14–100], *p* = 0.02), and lower fibrinolysis speed (0.41 [0.0–1.4] vs. 1.6 mm/min [0.1–2.7], *p* = 0.01). In the composite ICU population, 61% (97/159) demonstrated prolonged lysis time indicating impaired fibrinolytic capacity. These patients had higher 30-day mortality (adjusted odds ratio [OR]: 2.26 [0.83–6.69]) and VTE risk (OR: 3.84 [0.87–17.8]) than patients with normal lysis time.

**Conclusion** Sepsis patients showed impaired fibrinolysis measured with ROTEM®-tPA compared with nonsepsis patients and ROTEM®-tPA lysis time was associated with 30-day mortality and VTE in the entire ICU cohort.

## Introduction

Impairment of the fibrinolytic system is increasingly considered a key contributor in sepsis-induced coagulopathy (SIC), which is a frequent and serious complication to sepsis. 1 SIC is characterised by global activation of the coagulation system with microvessel thrombosis and simultaneous consumption of coagulation factors leading to increased bleeding risk. Its presentation can range from subclinical coagulation derangements to disseminated intravascular coagulation (DIC) in the most severe cases. 2 The pathophysiology behind SIC is complex and not yet fully understood. However, it is increasingly apparent that dysregulated fibrinolysis may play a pivotal role in the development of SIC. 3 4 5 Thus, impaired fibrinolysis in sepsis may contribute to adverse outcomes such as microvascular thrombosis, organ dysfunction, and mortality. 6 7 8 9 10

Additionally, abnormalities in the fibrinolytic system have been described in other critically ill patient populations including trauma patients, 11 postoperative patients, 12 and individuals with liver disease. 13 Currently, we have few, if any, options for assessing fibrinolysis in the routine diagnostic laboratory. 14 Circulating plasma concentrations of pro- and antifibrinolytic proteins such as plasminogen activator inhibitor 1 and thrombin-activatable fibrinolysis inhibitor can be measured 14 but do not provide dynamic information about fibrinolytic capacity. Plasma-based clot formation and lysis assays are available for research use and can provide more detailed information on fibrinolytic capacity 9 15 16 ; but due to lack of automation and standardisation, these assays are currently not suitable for routine clinical use, especially not in critically ill patients where short turnaround times are needed.

In contrast, viscoelastic haemostatic tests, e.g., rotational thromboelastometry (ROTEM®), are implemented in routine laboratories or point-of-care settings worldwide and are well suited for the acute setting as they have short runtimes and are semi-automated. Furthermore, as the viscoelastic tests utilise whole blood, they enable a global evaluation of fibrinolysis that considers the influence of cellular components on the fibrinolytic process. However, the most widely used viscoelastic protocols are designed for use in bleeding patients and are not sensitive toward hypofibrinolysis as they include 0% lysis in their reference interval. Thus, the evaluation and quantification of impaired fibrinolysis in the clinical setting remains a challenge. 4

In recent years, viscoelastic assays modified with tissue plasminogen activator (tPA) or other plasminogen activators have been developed. 17 18 19 20 Exogenous tPA is added to stimulate fibrinolysis, which means that in contrast to standard viscoelastic tests, full lysis is obtained within the clinically relevant runtime of an hour. This allows the detection of hypofibrinolysis in the patient sample. These assays have been used in sepsis patients with promising results. 21 22 23 However, such studies have lacked critically ill nonsepsis control groups and only one study investigated associations between fibrinolytic status and clinical outcomes, such as organ failure and mortality. 21

Our research group has recently set up a modified ROTEM® with tPA based on standard EXTEM reagents. 24 The primary objective of the present study was to investigate fibrinolytic capacity using our novel ROTEM®-tPA assay in sepsis patients admitted to the intensive care unit (ICU) compared with nonsepsis ICU patients and healthy individuals. Moreover, we aimed to explore whether fibrinolytic impairment on the first day of admission was associated with organ failure, venous thromboembolism (VTE), or 30-day mortality in ICU patients both with and without sepsis.

## Materials and Methods

### Design and Study Population

The present study was designed as a prospective cohort study. Patients admitted to the ICU at the Aarhus University Hospital, Denmark from September 2022 to April 2023 were screened for eligibility. Adult patients (≥18 years old) with an expected length of ICU stay exceeding 12 hours were included. Patients with prior ICU admission within the preceding 3 months or who had received antifibrinolytic or fibrinolytic treatment within 24 hours before blood sampling were excluded. Patients were stratified based on the presence of sepsis at the time of blood sampling according to the Sepsis-3 guidelines. 25 In cases of uncertainty regarding the sepsis diagnosis, the authors determined the categorisation by assessing whether changes in the Sequential Organ Failure Assessment (SOFA) score were most likely attributable to the patient's infection or to other clinical conditions. A blood sample was collected within the first 24 hours of ICU admission, specifically on the routine morning rounds on the day following admission. Patients were exclusively included on weekdays for logistic reasons. Written informed consent was obtained following the blood sampling procedure from the patient or next of kin and from an independent ICU physician. Data on ROTEM®-tPA in healthy individuals were obtained from 38 blood donors enrolled from the Department of Clinical Immunology, Aarhus University Hospital. 24 The study was approved by the Central Denmark Region Committees on Health Research Ethics (file no. 1-10-72-162-20).

### Clinical Data

Clinical data were collected from the patients' electronic medical journals and ICU observation charts and managed using the REDCap electronic data capture tools hosted at the Aarhus University, Denmark. 26 27 At the time of study enrollment, information regarding age, sex, body mass index (BMI), smoking status, and comorbidities was recorded. Presence of septic shock was assessed in accordance with the Sepsis-3 guidelines. 25 Moreover, details on treatment prior to blood sampling were documented, including treatment with extracorporeal membrane oxygenation, renal replacement therapy, major surgeries, and the use of anticoagulant medication. Simplified Acute Physiology Score (SAPS) III was assessed by the attending ICU physician at the time of admission. 28 DIC scores were calculated according to the International Society for Thrombosis and Haemostasis (ISTH). 29 SIC scores 30 and the highest SOFA scores 25 on the day of blood sampling were also evaluated.

During the 30 days following ICU admission, data were prospectively collected regarding length of ICU stay, administration of vasopressor medications, utilisation of renal replacement therapy or mechanical ventilation during admission, 30-day all-cause mortality, and VTE during the 30 days, verified by relevant imaging (e.g., ultrasound, computed tomography scan). Imaging was obtained at the discretion of the attending physician. Additionally, data regarding bleeding (World Health Organization [WHO] Bleeding Score grade ≥ 1) 31 during the first 7 days of ICU admission were obtained.

### Laboratory Methods

#### Rotational Thromboelastometry Modified with Tissue Plasminogen Activator

Blood was drawn from intra-arterial catheters or, if such a catheter was not in place, by venipuncture with minimal stasis, and collected in citrated tubes (1.8 mL BD Vacutainer 3.2% sodium citrate). The collected samples were gently inverted and allowed to rest for 30 minutes. The ROTEM®-tPA assay was performed as previously described. 24 Briefly, undiluted EXTEM reagent was used as the tissue factor (TF) source, and STARTEM reagent as the calcium source, similar to the standard EXTEM protocol. Human recombinant tPA (Calbiochem, Sigma-Aldrich, Merck, Darmstadt, Germany) was added to the STARTEM reagent immediately before analysis to achieve a final concentration of 125 ng/mL tPA in the cup. The ROTEM® analysis was performed at 37°C with a runtime of 60 minutes. All analyses were run in duplicate and mean parameter values were used.

**Fig. 1:**
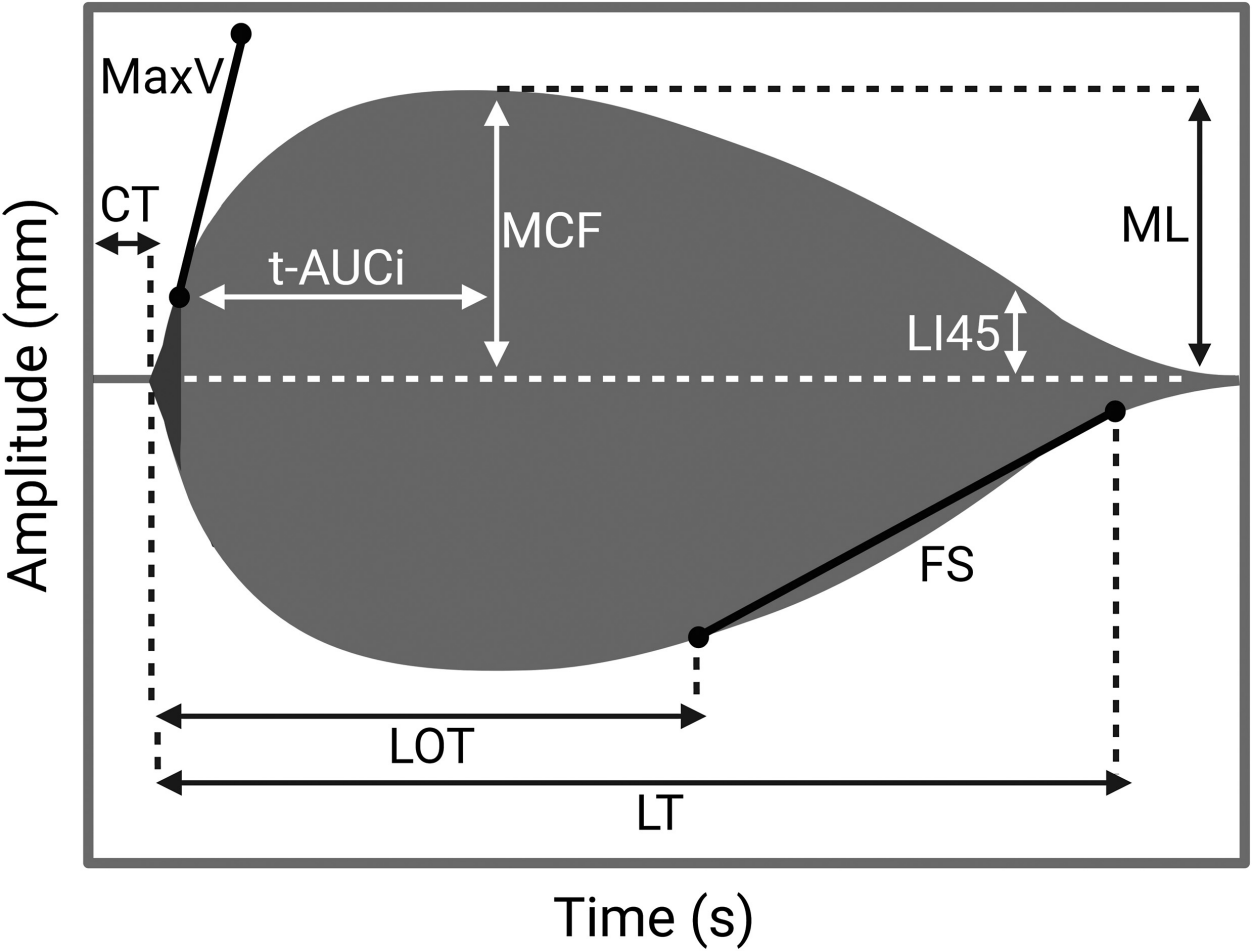
ROTEM® coagulation and lysis parameters. CT, clotting time (seconds, time until an amplitude of 2 mm is reached); FS, fibrinolysis speed (mm/min, clot breakdown speed in mm/min between LOT and LT; LI45, lysis index 45 (% of clot amplitude of MCF at 45 minutes after CT); LOT, lysis onset time (seconds, time from CT to 15% decrease in amplitude of MCF); LT, lysis time (seconds, time from CT until clot firmness has decreased to 10% of MCF); MaxV, maximum velocity (mm/min, maximum clot formation speed); MCF, maximum clot firmness (mm, the maximum amplitude reached); ML, maximum lysis (%, maximum lysis detected during the runtime); t-AUCi, time to attain maximal clot amplitude after reaching maximal clot formation velocity (minutes, time from MAXV-t to MCF-t).

For each channel, the following standard ROTEM® parameters were registered: clotting time (CT, seconds), maximum clot firmness (MCF, mm), maximum velocity (MaxV, mm/min), lysis index 45 (LI45, %), maximum lysis (ML, %), lysis onset time (LOT, seconds), and lysis time (LT, seconds). ROTEM® parameters are depicted in Fig. 1 . Patients who did not achieve an LOT or an LT during the 60-minute runtime due to hypofibrinolysis were assigned an LOT and/or an LT of 3,600 seconds.

Time to attain maximal clot amplitude after reaching maximal clot formation velocity (t-AUCi, min) was computed according to the methodology by Scarlatescu et al using the formula: t-AUCi = (MCF-t + CT) − MaxV-t. 23

Fibrinolysis speed (FS, mm/min), the clot breakdown speed between LOT and LT, was calculated according to the approach for FS _c_ outlined by Kuiper et al 22 using the formula: FS = Δamplitude (LT − LOT)/Δtime (LT − LOT). In cases where no LT was available due to hypofibrinolysis, FS was calculated using the amplitude at 60 minutes:  . If an LOT was unavailable due to hypofibrinolysis, the patient was assigned an FS of 0 mm/min.

#### Routine Laboratory Analyses

Platelet count, activated partial thromboplastin time (aPTT), international normalised ratio (INR), antithrombin, fibrinogen, arterial lactate, and routine laboratory markers of inflammation and organ dysfunction were analysed at the automated routine laboratory at the Department of Clinical Biochemistry according to ISO15189:2021 accredited protocols. Leukocyte count and platelet count were analysed on Sysmex XN-9000 (Sysmex, Kobe, Japan). INR (Medirox Owren's PT reagent), aPTT (Siemens Dade Actin FS reagent), fibrinogen (functional, Clauss, Siemens Dade thrombin reagent), antithrombin (functional, Siemens INNOVANCE reagent) were analysed on Sysmex C5100 (Sysmex, Kobe, Japan). Arterial lactate was analysed on automated blood gas analysers (ABL800 and ABL90, Radiometer, Brønshøj, Denmark) and markers of inflammation and organ dysfunction were analysed on Siemens Atellica chemistry and immunochemistry analysers (Siemens Healthineers, Duisburg, Germany).

### Statistical Analysis

The primary endpoint was the difference in LT between sepsis and nonsepsis patients. Our protocol was developed to yield LTs of 40 minutes in healthy individuals with an assumed standard deviation (SD) of 10% (4 minutes). 24 We expected SD to be larger in an ICU population. With a study power (1 − β) of 0.9, a significance level (2α) of 0.05, a minimal relevant difference of 25%, and an estimated SD in ICU patients of double that of healthy individuals (i.e., 8 minutes), 23 sepsis patients and 23 nonsepsis patients had to be included.

Since we found impaired fibrinolysis not only in sepsis patients but also in nonsepsis ICU patients, we then defined a secondary endpoint: to investigate the association between LT and mortality in the overall ICU cohort.

Normal distribution was assessed visually with quantile–quantile plots. Continuous data were described using median and interquartile range for uniformity, since the majority of data did not follow a Gaussian distribution. Categorical data were presented as numbers and percentages. Differences in continuous variables between groups were tested with the Mann–Whitney test, and Fisher's exact test was used for categorical data. Association between whole-blood fibrinolysis parameters and mortality was analysed using uni- and multivariate logistic regression and with the Kaplan–Meier method and the log-rank test.

All statistical analyses and graphs were generated using GraphPad Prism version 9.5.0.730 for Windows (GraphPad Software, San Diego, California, United States).

## Results

### Patient Characteristics

A total of 310 patients were screened for eligibility. Of these, 62 were excluded due to logistical reasons, 61 were excluded due to ICU admission within the preceding 3 months, 12 were excluded due to treatment with pro- or antifibrinolytic agents, 10 had admissions of less than 12 hours, and 6 did not consent to participate. Ultimately, 159 patients were enrolled, 30 sepsis patients and 129 nonsepsis patients.

Baseline clinical characteristics and data regarding bleeding, VTE, and mortality in sepsis versus nonsepsis patients are reported in Table 1 .

**Table 1:** Clinical characteristics and bleeding, venous thromboembolism, and mortality data of sepsis and nonsepsis patients

Characteristics	Sepsis patients ( *n* = 30)	Nonsepsis patients ( *n* = 129)
**SAPS III score at admission**	63 (48–79)	60 (46–73)
**SOFA score day 1**	9 (7–11)	9 (5–11)
**Arterial lactate at admission (mmol/L)**	1.7 (1.0–2.8)	1.6 (1.1–3.5)
**DIC score**	3 (2–4)	2 (2–3)
**DIC score ≥ 5**	6 (20%)	5 (4%)
**SIC score**	3 (2–4)	–
**Septic shock**	16 (53%)	–
**Occurrence of VTE (within 30 d)**	6 (20%)	7 (5%)
**Any bleeding, WHO bleeding score ≥1 (within 7 d)**	21 (70%)	89 (69%)
**Mortality (within 30 d)**	12 (40%)	25 (19%)

Abbreviations: DIC, disseminated intravascular coagulation; ICU, intensive care unit; SAPS, Simplified Acute Physiology Score; SIC, sepsis-induced coagulopathy; SOFA, Sequential Organ Failure Assessment; VTE, venous thromboembolism; WHO, World Health Organization.

Notes: Continuous variables are reported as medians (interquartile range), whereas categorical variables are presented as numbers and percentages. The following parameters have missing data: arterial lactate at admission ( *n* = 2).

Sepsis patients and nonsepsis patients had comparable SAPS III scores, SOFA scores, and arterial lactate at ICU admission, but sepsis patients demonstrated higher DIC scores (3 [2–4] vs. 2 [2–3] than nonsepsis patients. Among the sepsis patients, 16 (53%) had septic shock.

Sepsis patients experienced significantly worse outcomes in terms of 30-day mortality (40 vs. 19%) and VTE development (20 vs. 5%) compared with nonsepsis patients. However, both groups showed a similar incidence of bleeding episodes during the first 7 days of admission (70 vs. 69%).

### ROTEM®-tPA in Sepsis and Nonsepsis Patients

**Fig. 2:**
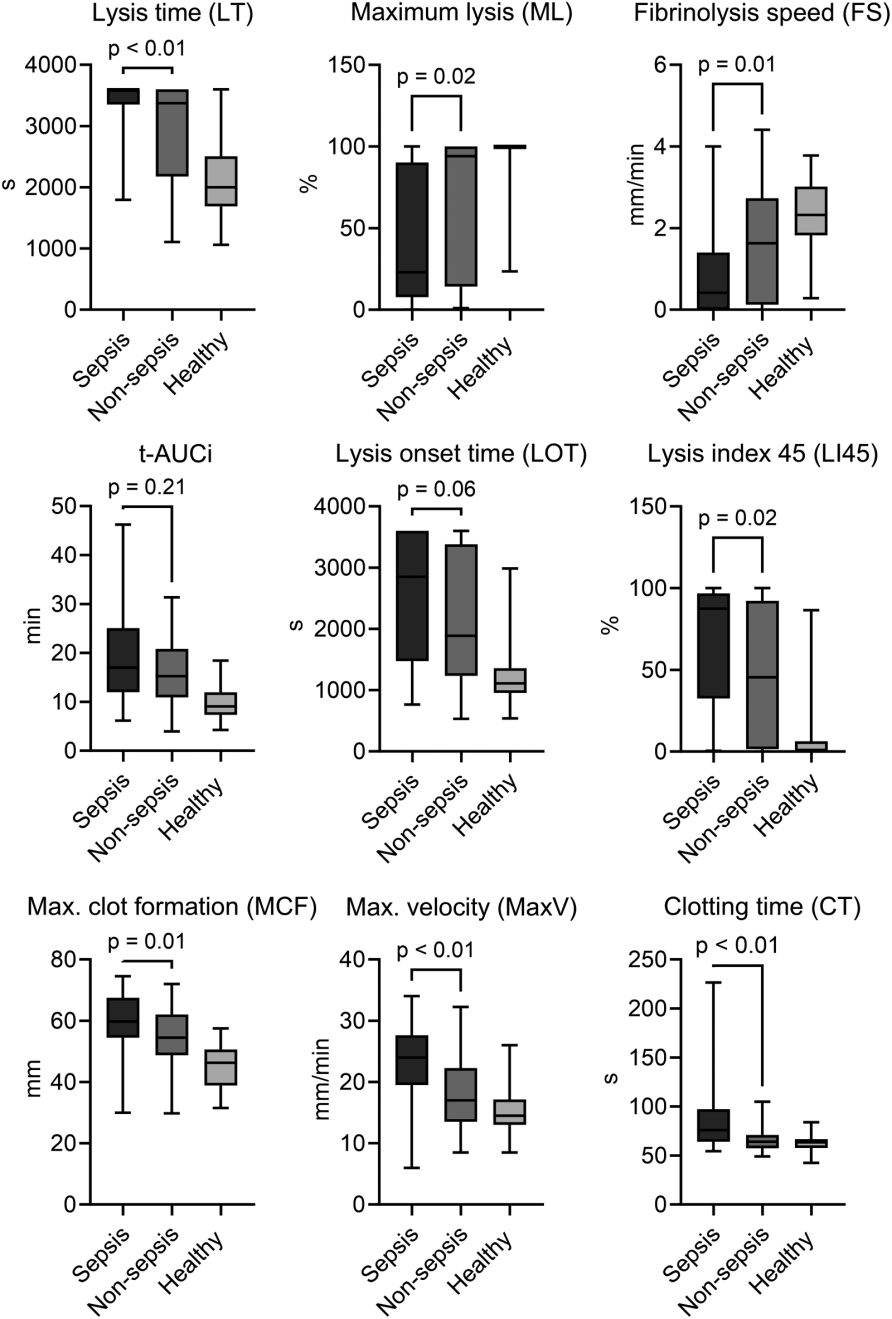
Coagulation and fibrinolysis parameters measured by ROTEM®-tPA in sepsis patients, nonsepsis patients, and healthy individuals. Boxes represent medians with interquartile ranges, and whiskers represent the 2.5 and the 97.5 percentiles. *p* -values were calculated with the Mann–Whitney test. t-AUCi, time to attain maximal clot amplitude after reaching maximal clot formation velocity.

**Fig. 3:**
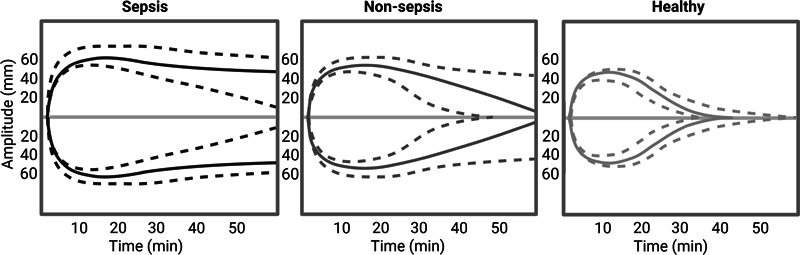
Illustrations of ROTEM®-tPA tracings for sepsis patients, nonsepsis patients, and healthy individuals. Median ROTEM® traces are depicted as solid lines and dotted lines represent the interquartile range. Figure created with Biorender.com.

Results of the ROTEM®-tPA analysis are shown in Figs. 2 and 3 . Sepsis patients differed from nonsepsis patients across all ROTEM® parameters and were characterised by a markedly impaired fibrinolytic capacity compared with both nonsepsis patients and healthy individuals. Median LT was significantly prolonged among sepsis patients compared with nonsepsis patients (3,600 [3,352–3,600] vs. 3,374 seconds [2,175–3,600], *p* < 0.01). A larger proportion of sepsis patients displayed LTs above the 97.5 percentile of the 38 healthy individuals (3,000 seconds 24 compared with nonsepsis patients ( *n* = 24, 80% vs. *n* = 73, 57%). Overall, only very few patients demonstrated increased fibrinolysis, as no sepsis patients and two nonsepsis patients had an LT below the 2.5 percentile of the 38 healthy individuals (1,080 seconds). 24 ML was significantly lower in sepsis patients than in nonsepsis patients (23 [8–90] vs. 94 [14–100] %, *p* = 0.02), as was FS (0.41 [0.0–1.4] vs. 1.6 [0.1–2.7] mm/min, *p* = 0.01). t-AUCi was higher in sepsis patients than nonsepsis patients, although the difference was not statistically significant (17 [12–25] vs. 15 [11–21] minutes, *p* = 0.21). Furthermore, the sepsis population showed prolonged LOT as well as higher LI45, all indicative of hypofibrinolysis.

Within the nonsepsis group, fibrinolytic capacity ranged from normal to severely impaired. Altogether, nonsepsis patients had substantially prolonged LOT, LT, and t-AUCi as well as decreased ML and FS compared with healthy individuals. Thus, the nonsepsis group were characterised by impaired fibrinolysis when compared with healthy individuals, although not as pronounced as the sepsis group.

Regarding coagulation parameters, the sepsis group exhibited elevated MCF as well as a higher MaxV compared with nonsepsis patients, indicating increased clot strength. However, sepsis patients also demonstrated a slightly longer median CT compared with controls as shown in Fig. 2 .

### Association between Clinical Outcomes and Impaired Fibrinolysis

All 159 patients, including both sepsis and nonsepsis cases, were stratified according to the presence of hypofibrinolysis defined as LT above the 97.5 percentile of 38 healthy individuals as previously calculated (50 minutes). 24 Most patients (61%) displayed LTs above the 97.5 percentile, indicating an impaired fibrinolytic capacity, whereas the remaining patients (39%) had LTs below the 97.5 percentile, indicating normal fibrinolysis. Patients with LT below the 2.5 percentile ( *n* = 2) was included in the normal LT group. Clinical and outcome data for patients with normal and impaired fibrinolysis are presented in Table 2 .

**Table 2:** Demographics, clinical characteristics, and bleeding, venous thromboembolism, and mortality data of intensive care unit patients with normal and impaired fibrinolysis

Characteristics	Impaired fibrinolysis ( *n* = 97)	Normal fibrinolysis ( *n* = 62)	*p*
**Female sex**	61 (37%)	41 (34%)	–
**Age (y)**	67 (54–74)	63 (56–71)	–
** BMI (kg/m ^2^ ) **	27 (24–31)	25 (23–30)	–
**Current smoker**	29 (29%)	16 (32%)	–
**Days in hospital before ICU admission**	0 (0–2)	0 (0–1)	–
**Sepsis on day 1**	24 (25%)	6 (10%)	–
**SAPS III score at admission**	63 (49–76)	59 (44–71)	0.15
**SOFA score day 1**	10 (7–11)	7 (4–10)	<0.01
**Arterial lactate at admission (mmol/L)**	1.9 (1.1–3.9)	1.4 (1.0–2.3)	<0.01
**DIC score day 1**	2 (2–3)	2 (2–3)	0.42
**DIC score ≥ 5**	9 (9%)	2 (3%)	0.20
**Interventions before blood sampling**			
**Renal replacement therapy (24 h)**	5 (5%)	0 (0%)	–
**ECMO (24 h)**	9 (9%)	1 (2%)	–
**Major surgery (7 d)**	32 (33%)	7 (12%)	–
**Platelet inhibitors (14 d)**	32 (33%)	25 (40%)	–
**Vitamin K antagonists (14 d)**	7 (7%)	1 (2%)	–
**Direct oral anticoagulants (3 d)**	8 (8%)	13 (21%)	–
**Heparins (24 h)**			
**Low molecular weight, prophylactic dose**	22 (23%)	12 (19%)	–
**Low molecular weight, therapeutic dose**	15 (15%)	5 (8%)	–
**Unfractionated**	15 (15%)	5 (8%)	–
**No heparin**	45 (46%)	40 (65%)	–
**Comorbidities**			
**Hypertension**	40 (41%)	29 (43%)	–
**Diabetes**	21 (22%)	16 (26%)	–
**Ischaemic heart disease**	18 (19%)	19 (31%)	–
**Solid cancer**	12 (12%)	4 (6%)	–
**Haematologic cancer**	7 (7%)	3 (5%)	–
**S** ARS **-Cov-2**	1 (1%)	1 (2%)	–
**Occurrence of VTE (within 30 d)**	11 (11%)	2 (3%)	0.08
**Length of ICU stay (d)**	4 (2–8)	2 (1–5)	0.03
**Interventions during ICU admission**			
**Mechanical ventilation**	79 (81%)	33 (53%)	<0.001
**Renal replacement therapy**	15 (16%)	3 (5%)	0.04
**Vasopressor treatment**	90 (93%)	39 (63%)	<0.0001
**Any bleeding, WHO bleeding score ≥1 (within 7 d)**	75 (77%)	35 (57%)	<0.01
**Mortality (within 30 d)**	30 (31%)	7 (11%)	<0.01

Abbreviations: BMI, body mass index; DIC, disseminated intravascular coagulation; ECMO, extracorporeal membrane oxygenation; ICU, intensive care unit; SAPS, Simplified Acute Physiology Score; SARS-Cov-2, severe acquired respiratory syndrome coronavirus-2; SIC, sepsis-induced coagulopathy; SOFA, Sequential Organ Failure Assessment; VTE, venous thromboembolism; WHO, World Health Organization.

Notes: Continuous variables are reported as medians (interquartile range) while categorical variables are presented as numbers and percentages. The following parameters have missing data: BMI ( *n* = 8), arterial lactate at admission ( *n* = 2) and length of ICU stay ( *n* = 2). Patients who died during ICU admission ( *n* = 29) were excluded from the length of stay calculation. Further, there are missing SOFA score component values on two patients and a score of 0 was imputed for missing variables.

**Table 3:** Laboratory parameters of intensive care unit patients on day 1 of intensive care unit admission

Parameter	Impaired fibrinolysis ( *n* = 97)	Normal fibrinolysis ( *n* = 62)
** Platelet count (10 ^9^ /L) **	221 (160–277)	209 (165–273)
**D-Dimer (mg/L FEU)**	3.0 (1.1–7.3)	3.9 (1.1–7.6)
**International normalised ratio, INR**	1.2 (1.1–1.3)	1.2 (1.1–1.3)
**Activated partial thromboplastin time, aPTT (s)**	27 (24–33)	26 (23–30)
**Antithrombin (µmol/** L **)**	0.80 (0.66–0.92)	0.87 (0.76–1.0)
**Fibrinogen (µmol/** L **)**	12.0 (8.7–15.9)	11.6 (8.4–14.7)
**C-reactive protein, CRP (mg/L)**	62 (40–194)	56 (16–139)
** Leukocyte count (10 ^9^ /L) **	13.1 (10.4–17.2)	10.0 (7.8–14.1)

Abbreviation: FEU, fibrinogen equivalent unit.

Notes: Reported as medians (interquartile range). The following parameters have missing data: C-reactive protein ( *n* = 1), leukocyte count ( *n* = 1).

Patients with normal fibrinolysis and impaired fibrinolysis had comparable ages, BMI, sex distribution, DIC scores, and SAPS III scores, but patients with impaired fibrinolysis had higher SOFA scores and higher arterial lactate than patients with normal fibrinolysis.

A larger proportion in the normal fibrinolysis group had ischaemic heart disease than in the impaired fibrinolysis group and cancer was slightly more prevalent among those with impaired fibrinolysis than those with normal fibrinolysis. Apart from that, the groups were comparable with regards to comorbidity prevalence.

Regarding biochemical parameters obtained on the first morning of admission ( Table 3 ), patients with impaired fibrinolysis demonstrated higher C-reactive protein and leukocyte count than those with normal fibrinolysis. The groups were, however, comparable with regards to platelet count, D-Dimer, INR, aPTT, antithrombin, and fibrinogen.

All-cause 30-day mortality was 23%. In univariate analysis, age, SAPS-III score, SOFA score, DIC score, and day 1 ROTEM®-tPA parameters LT, LOT, ML, FS, LI45, and t-AUCi fibrinolysis were associated with 30-day mortality ( Table 4 ). Impaired fibrinolysis on day 1, defined as LT > 50 minutes, was strongly associated with 30-day mortality, odds ratio [OR] = 3.52 (95% confidence interval [CI]: 1.51–9.27). This same pattern was also observed when exclusively looking at mortality among the 129 nonsepsis patients, OR = 3.85 (95% CI: 1.33–9.88). SOFA-score and SAPS-score had the best overall predictive ability for 30-day mortality (area under receiver operator characteristics [ROC] curve 0.79 and 0.77), whereas ROTEM®-tPA parameters yielded very moderate areas under ROC curve, highest for t-AUCi (0.67).

**Table 4:** Odds ratios for 30-day mortality with univariate logistic regression

Parameter	OR (95% CI)	*p*	AUROC
**Age, per 1** - **y increase**	1.06 (1.02–1.09)	<0.01	0.70
**Female sex**	0.82 (0.37–1.77)	0.62	0.52
**SAPS III, pr. 1** - **point increase**	1.07 (1.04–1.10)	<0.0001	0.77
**SOFA score, pr. 1** - **point increase**	1.43 (1.25–1.67)	<0.0001	0.79
**ISTH DIC score, pr. 1** - **point increase**	1.62 (1.19–2.27)	<0.01	0.62
** Impaired fibrinolysis a**	3.52 (1.51–9.27)	<0.01	0.63
**LT, pr. 1** - **min increase**	1.05 (1.01–1.09)	0.01	0.62
**LOT, pr. 1** - **min increase**	1.03 (1.01–1.05)	0.01	0.63
**ML, pr. 10% increase**	0.91 (0.83–0.99)	0.03	0.66
**FS, pr. 1** - **mm/min increase**	0.74 (0.55–0.99)	0.05	0.62
**LI45, pr. 1% increase**	1.01 (1.00–1.02)	0.01	0.66
**MCF, pr. 1** - **mm increase**	1.04 (1.00–1.08)	0.07	0.61
**MaxV, pr. 1** - **mm/min increase**	1.02 (0.97–1.08)	0.45	0.54
**t-AUCi, pr. 1** - **min increase**	1.09 (1.04–1.15)	<0.001	0.67

Abbreviations: AUROC, area under receiver operator characteristics curve; CI, confidence interval; DIC, disseminated intravascular coagulation; FS, fibrinolysis speed; ISTH, International Society on Thrombosis and Haemostasis; LI45, lysis index at 45 minutes; LOT, lysis onset time; LT, lysis time; MaxV, maximum velocity; MCF, maximum clot formation; ML, maximum lysis; OR, odds ratio; SAPS, Simplified Acute Physiology Score; SOFA, Sequential Organ Failure Assessment; t-AUCi, time to attain maximal clot amplitude after reaching maximal clot formation velocity.

a Defined as LT > 50 minutes.

**Fig. 4:**
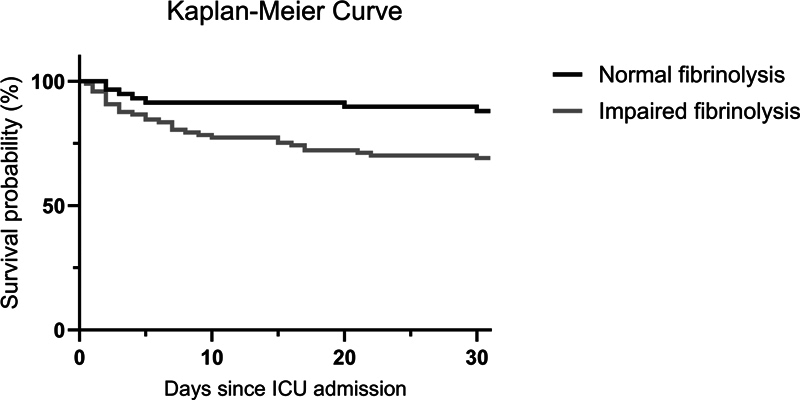
Probability of survival in 62 patients with normal lysis times (≤50 minutes) and 97 patients with prolonged lysis times (>50 minutes). ICU, intensive care unit.

In multivariate analysis including SAPS-III score at admission, SOFA-score, DIC-score, and impaired fibrinolysis (LT > 50 minutes; Table 5 ), impaired fibrinolysis was still associated with 2.3 times higher risk of 30-day mortality, although the association was no longer statistically significant (OR = 2.26 [95% CI: 0.83–6.69]). Areas under ROC curve were similar for a model including SAPS + SOFA + DIC score (0.83 [95% CI: 0.75–0.89]), a model including SAPS + SOFA + DIC + impaired fibrinolysis (0.83 [95% CI: 0.75–0.90]), and a model including SAPS + SOFA + DIC + t-AUCi (0.83 [95% CI: 0.75–0.90]). A Kaplan–Meier analysis revealed distinct differences in survival ( *p* = 0.009), as shown in Fig. 4 , with a 30-day survival probability of 69% in the impaired fibrinolysis group and 89% in the normal fibrinolysis group.

**Table 5:** Odds ratio for 30-day mortality with a multivariate logistic regression model

Parameter	OR (95% CI)	*p*
**SAPS III score, pr. 1** - **point increase**	1.04 (1.01–1.08)	0.01
**SOFA score, pr. 1** - **point increase**	1.28 (1.08–1.54)	<0.01
**ISTH DIC score, pr** . **1** - **point increase**	0.94 (0.61–1.44)	0.77
** Impaired fibrinolysis a**	2.26 (0.83–6.69)	0.12

Abbreviations: CI, confidence interval; DIC, disseminated intravascular coagulation; ISTH, International Society on Thrombosis and Haemostasis; LT, lysis time; OR, odds ratio; SAPS, Simplified Acute Physiology Score; SOFA, sequential organ failure assessment.

a Defined as LT > 50 minutes.

Furthermore, patients with impaired fibrinolysis on day 1 had a higher incidence of symptomatic VTE within 30 days of ICU admission ( *n* = 11/97, 11%) than those with normal fibrinolysis ( *n* = 2/62, 3%), with an OR of 3.84 (95% CI: 0.87–17.8), *p* = 0.08. Impaired fibrinolysis was also associated with longer ICU stays and with more interventions during ICU admission. A higher incidence of bleeding episodes with the WHO bleeding score ≥ 1 was observed in patients with impaired fibrinolysis than in patients with normal fibrinolysis (75 [77%] vs. 35 [57%]).

## Discussion

The main finding of the present study was a significant impairment of whole-blood fibrinolytic capacity in sepsis patients compared with nonsepsis ICU patients measured with ROTEM®-tPA. Further, ICU patients generally had impaired fibrinolysis compared with healthy individuals, regardless of the presence of sepsis. In the entire ICU cohort, patients with impaired fibrinolysis on day 1 of their ICU admission had a three times increased risk of venous thrombosis and close to three times higher 30-day mortality compared with patients with normal fibrinolysis. Our findings underline the importance of fibrinolysis in critical illness and establish ROTEM®-tPA as a clinically relevant and feasible assay for assessment of fibrinolysis in critically ill patients.

Recent studies utilising plasma-based clot lysis assays have reported an impaired fibrinolytic capacity in the majority of sepsis patients compared with nonsepsis patients or healthy individuals. 13 15 32 Our findings expand previous studies by using a whole-blood assay with a large nonsepsis ICU control group. Apart from the current study, three other studies have used modified viscoelastic tests in patients with sepsis. Kuiper et al used ROTEM® modified with tPA in 21 sepsis patients but excluded those with DIC and did not investigate associations with clinical endpoints. They reported impaired fibrinolytic capacity in sepsis patients compared with healthy individuals, pregnant women, and cirrhotic liver disease patients. 22 Panigada et al used a urokinase (uPA) and kaolin-activated thromboelastography (UK-TEG) assay to compare fibrinolysis in 40 sepsis patients with healthy individuals and found a higher TEG maximal amplitude and impaired fibrinolysis indicated by lower TEG lysis index at 30 minutes in sepsis patients. They also investigated associations between fibrinolysis impairment and adverse outcomes in sepsis patients and reported higher SOFA scores, mortality, and longer ICU stays in patients classified as “low responders” to urokinase. 21 A recent paper by the ISTH SSC (Scientific and Standardization Committee) subcommittee on fibrinolysis by Scarlatescu et al 23 evaluated the utility of t-AUCi parameter (time between MaxV and maximum clot formation) in ROTEM® with different concentrations of tPA. In 30 sepsis patients, they found that t-AUCi could discriminate between sepsis patients and healthy controls and was associated with LI45. We also found higher t-AUCi in sepsis patients than in healthy controls, but t-AUCi did not differ significantly between sepsis and nonsepsis ICU patients.

The present study and Kuiper et al 22 found impaired fibrinolytic capacity in the majority of sepsis patients, whereas Panigada et al 21 reported that more than half of sepsis patients exhibited a normal response to uPA in their UK-TEG assay. This discrepancy may stem from differences in assays, most importantly the use of kaolin instead TF and uPA instead of tPA, or use of different parameters and cutoff points for the definition of hypofibrinolysis.

Since tPA- or uPA-modified viscoelastic assays are relatively new, the most appropriate parameters for describing fibrinolytic capacity with these assays have not yet been defined, nor have normal ranges or reference intervals been established. The significant differences in outcomes observed in the present study suggest that our definition of hypofibrinolysis has clinical relevance. However, other parameters than LT may yield more details—for instance LOT, ML, or the calculated FS. Scarlatescu et al proposed t-AUCi to assess sepsis-related hypofibrinolysis, but since we found no significant difference between sepsis and nonsepsis ICU patients, other parameters might be better suited for this. However, the discrepancy between our results may be due to different tPA concentrations between our assays or differences between our included sepsis populations.

We observed that impaired fibrinolytic capacity correlated with organ dysfunction and decreased survival in the composite ICU population. Of note, we found that the association between hypofibrinolysis and mortality persisted even after excluding sepsis patients from the analysis, indicating that the association cannot be explained by the higher mortality rates observed in the sepsis group. However, the addition of LT to a model including SAPS-III, SOFA score and DIC score did not improve area under ROC curve. Associations between mortality and impaired fibrinolysis measured with tPA-modified TEG have also been reported in other patient groups such as haemorrhagic shock patients 20 and liver transplant recipients, 33 which supports the clinical relevance of viscoelastic tests with tPA.

To the best of our knowledge, we are the first to investigate the association between venous thrombosis and hypofibrinolysis using a modified viscoelastic assay. Previous studies have reported an association between thrombosis and hypofibrinolysis assessed with plasma-based assays. 34 Our findings revealed an OR of more than 3 for VTE development in ICU patients with impaired fibrinolysis compared with those with normal fibrinolysis. Although the wide confidence interval limits the precision of our estimate, our findings indicate a substantial increase in risk of VTE associated with hypofibrinolysis and highlight the potential of ROTEM®-tPA as a marker for identifying patients at increased risk of VTE.

One unexpected result was a higher 7-day incidence of bleeding episodes with a WHO bleeding score ≥ 1 in patients with impaired fibrinolysis compared with normal fibrinolysis. A possible explanation for this observation is that the impaired fibrinolysis group experienced significantly longer ICU stays, underwent more invasive procedures, and received more heparins during ICU admissions.

Few patients showed increased fibrinolysis compared with healthy individuals, which may in part be ascribed to the exclusion of those who were treated with tranexamic acid due to a possible hyperfibrinolytic state.

Strengths of the present study include a large and diverse ICU group and systematic collection of detailed clinical and biochemical information. However, this study has certain limitations that should be considered. First, this was a single-center study with a relatively small sepsis group ( *n* = 30) and to increase the generalisability of our findings, validation in larger cohorts is needed. Further, due to the relatively infrequent event rates for mortality, VTE, and other outcomes, it was not possible to perform statistical analysis to adjust for possible confounding factors. As the blood sample was taken on the morning round, the timing of blood sampling in relation to ICU admission was variable between 1 and 23 hours. Despite this variability, we still found a clear association between ROTEM®-tPA results and clinical outcomes. Furthermore, this design had the advantage of minimising circadian variation.

Lastly, although our findings demonstrate associations between impaired fibrinolysis and adverse outcomes in ICU patients, we have not demonstrated causality. Further research should focus on underlying mechanisms behind lysis resistance assessed in whole blood and the specific contributions from both pro- and antifibrinolytic proteins and the formed elements of blood to identify potential therapeutic targets.

In summary, our findings demonstrate that ROTEM®-tPA is a sensitive and feasible tool for investigation of fibrinolysis in critically ill patients. Impaired fibrinolysis assessed with ROTEM®-tPA was associated with both mortality and VTE risk in ICU patients. This method holds the potential to improve early diagnosis and management of disturbed fibrinolysis with the perspective of more individualised therapy.

## Notes

**What is Known on this Topic?**

- Dysregulated fibrinolysis may play a pivotal role in the development of sepsis-induced coagulopathy.
- Routine laboratory tests for fibrinolysis are currently very limited.
- Impact of fibrinolytic capacity on clinical outcome in intensive care patients is poorly investigated.

**What Does this Paper Add?**

- We used rotational thromboelastometry® modified with tissue plasminogen activator ((ROTEM®-tPA), a novel fibrinolysis assay, to investigate fibrinolysis in 159 intensive care (ICU) patients.
- ROTEM®-tPA showed impaired fibrinolysis in sepsis patients compared with non-sepsis patients.
- Impaired fibrinolysis was associated with higher 30-day mortality and venous thromboembolism risk in the entire ICU cohort.
